# Changes in Coagulation Parameters and Metabolic Profile in Hospitalized Patients with Metabolic Dysfunction-Associated Steatohepatitis Receiving Vitamin K: A Retrospective Observational Study

**DOI:** 10.3390/clinpract16050097

**Published:** 2026-05-21

**Authors:** Magdalena Lixandru, George Maniu, Cosmin Ionut Lixandru, Florin Grosu

**Affiliations:** 1 Faculty of Medicine, “Lucian Blaga” University of Sibiu, 550169 Sibiu, Romania; cosmin.lixandru@ulbsibiu.ro (C.I.L.); florin.grosu@ulbsibiu.ro (F.G.); 2 Sibiu County Emergency Clinical Hospital, 550245 Sibiu, Romania; 3 Valcea County Emergency Hospital, 240284 Valcea, Romania; 4 Mathematics and Informatics Department, Research Center in Informatics and Information Technology, Faculty of Sciences, “Lucian Blaga” University of Sibiu, 550025 Sibiu, Romania

**Keywords:** MASH, vitamin K, non-alcoholic steatohepatitis, coagulopathy, metabolic dysfunction

## Abstract

**Background/Objectives**: Metabolic dysfunction-associated steatohepatitis (MASH) is frequently accompanied by disturbances in coagulation and metabolic homeostasis, partly related to impaired handling of vitamin K-dependent pathways. Although vitamin K is often administered to correct abnormal coagulation tests, its biochemical impact in hospitalized patients with MASH remains insufficiently characterized. This study aimed to evaluate changes in coagulation and metabolic parameters in hospitalized patients with MASH receiving vitamin K supplementation. **Methods**: We conducted a retrospective study of 84 hospitalized MASH patients who received vitamin K supplementation. Biochemical parameters were recorded at admission and discharge to assess short-term changes during hospitalization. **Results**: Vitamin K supplementation was associated with modest changes in coagulation parameters, including reductions in PT, INR, and aPTT (e.g., PT decreased from 13.00 s to 11.00 s). Small numerical changes in transaminases, fasting glucose, and total cholesterol were observed during hospitalization, with limited clinical relevance. These patterns were comparable across fibrosis stages, with no significant differences between groups. **Discussion**: The observed biochemical findings are likely in-hospital factors rather than a direct metabolic effect of vitamin K. **Conclusions**: Vitamin K supplementation was associated with modest changes in coagulation parameters and small, clinically negligible variations in selected metabolic markers in patients with MASH, irrespective of fibrosis stage. These findings suggest a supportive biochemical effect in selected contexts; further prospective studies are needed to clarify their clinical relevance.

## 1. Introduction

Metabolic dysfunction-associated steatohepatitis (MASH), previously known as non-alcoholic steatohepatitis (NASH), represents the progressive inflammatory phenotype of metabolic dysfunction-associated steatotic liver disease (MASLD). It is characterized by hepatic steatosis, hepatocellular ballooning, lobular inflammation, and varying degrees of fibrosis, typically occurring in individuals with insulin resistance, obesity, and type 2 diabetes mellitus [1,2]. MASLD has become the leading cause of chronic liver disease in industrialized countries, with a global prevalence of around one quarter to one third of adults, while the progressive steatohepatitic form (historically termed NASH, now MASH) affects a smaller but clinically significant fraction of the population [3,4]. Beyond hepatic injury, this condition is strongly associated with extrahepatic metabolic disorders and cardiovascular complications, contributing to increased morbidity and mortality [5,6,7]. The rising burden of metabolic liver disease underscores the need for therapeutic strategies that address both hepatic and systemic metabolic dysfunction [8].

Vitamin K is a fat-soluble micronutrient essential for hepatic and extrahepatic physiology, serving as a cofactor for γ-glutamyl carboxylase and enabling the activation of vitamin K-dependent proteins, including coagulation factors II, VII, IX, and X, as well as the natural anticoagulants protein C and protein S [9]. Because the liver plays a central role in the vitamin K cycle—encompassing uptake, storage, and recycling—hepatic dysfunction may impair the activation of these proteins and contribute to coagulation abnormalities [10]. In addition, vitamin K metabolism is closely linked to lipid handling and bile-dependent absorption, processes that may be altered in metabolic liver disease [11]. Subclinical vitamin K deficiency has been described in chronic liver disease and may manifest as impaired coagulation parameters [12]. In addition to its role in coagulation, vitamin K may also influence hepatocyte function, inflammation, and fibrogenesis in chronic liver disease. Experimental and clinical studies suggest that vitamin K-dependent pathways may influence hepatocyte survival, inflammation, and fibrogenesis in chronic liver disease [9,13,14]. In population studies, lower vitamin K status has also been associated with higher levels of systemic inflammatory markers and metabolic dysfunction [15]. These observations have raised interest in the potential relationship between vitamin K biology and metabolic liver disease [16]. Preclinical and clinical evidence suggest that the response to vitamin K administration in liver disease may vary depending on the underlying pathology and baseline vitamin K status. In chronic liver disease, vitamin K supplementation does not consistently correct INR or reduce bleeding risk, indicating that functional deficiency is not universal and that empiric administration may not benefit all patients [17,18]. Conversely, small observational studies in selected clinical settings have reported modest short-term changes in coagulation parameters after vitamin K administration [19,20]. In metabolic liver disease, observational data suggest a possible relationship between vitamin K status and the burden of fatty liver disease, providing a rationale to further investigate vitamin K-related pathways in this population [21]. However, clinical evidence regarding the therapeutic relevance of vitamin K in liver disease remains limited. Current international guidelines recommend selective rather than routine vitamin K administration, reflecting uncertainty about its clinical benefit in different patient populations [21]. Most available studies have focused on advanced cirrhosis, while comparatively little information exists for patients with metabolic liver disease, in whom alterations in lipid metabolism and nutrient handling may influence vitamin K status and biological response [20]. Moreover, randomized evidence is lacking regarding whether correction of vitamin K status in this population can meaningfully influence coagulation parameters or metabolic profiles.

Recent population-based analyses have reported an inverse association between dietary vitamin K intake and the prevalence of metabolic dysfunction-associated steatotic liver disease, although these findings remain observational and causality has not been established [16]. Consequently, further research is needed to clarify whether vitamin K supplementation is associated with changes in coagulation parameters and metabolic markers in this population. The present study was therefore designed to evaluate changes in coagulation and metabolic parameters in hospitalized patients with metabolic liver disease receiving vitamin K supplementation.

The present study was designed to evaluate changes observed in coagulation and metabolic parameters in patients receiving vitamin K supplementation. The main objective was to explore whether vitamin K administration is associated with variations in coagulation function and overall metabolic status in this population. We hypothesized that vitamin K supplementation may be associated with changes in coagulation parameters in patients with metabolic liver disease. This approach aims to provide hypothesis-generating data regarding vitamin K-related biochemical dynamics in patients with MASH. Accordingly, this study represents a descriptive, real-world analysis of short-term biochemical changes observed during hospitalization, rather than an attempt to establish causality or therapeutic efficacy.

## 2. Materials and Methods

### 2.1. Study Design and Setting

This retrospective observational study was conducted in the Department of Internal Medicine, Vâlcea County Emergency Hospital, Râmnicu Vâlcea, Romania, between January 2020 and December 2024. The study analyzed clinical and laboratory data of patients hospitalized for metabolic liver disease and associated coagulation disturbances. Given the retrospective design and absence of a control group, the study was not intended to assess causality, but rather to describe associations observed in routine clinical practice. All data were retrieved from the hospital’s electronic medical records and laboratory database.

### 2.2. Patient Enrollment

The study included 84 adult patients aged between 18 and 85 years diagnosed with metabolic dysfunction-associated steatohepatitis (MASH). All patients received vitamin K supplementation during hospitalization as part of their therapeutic management for coagulation abnormalities. Liver function and coagulation parameters were periodically monitored, and only patients with sufficient follow-up data to assess treatment response and clinical evolution were included in the final analysis.

### 2.3. Diagnosis of MASH and Fibrosis Assessment

Inclusion criteria required a confirmed diagnosis of MASH established by imaging methods (ultrasound, CT, or MRI) and, when available, non-invasive tests such as FibroTest or transient elastography. Patients had to present an abnormal coagulation profile (elevated INR, prolonged prothrombin time, or thrombocytopenia) and biochemical evidence of hepatic dysfunction, including increased aminotransferases, total bilirubin, or documented fibrosis.

The study population also included individuals with overweight or obesity, defined according to international criteria (body mass index ≥ 25 kg/m^2^) or the presence of at least three components of the metabolic syndrome (hypertension, hyperglycemia, and dyslipidemia).

Exclusion criteria encompassed active viral (B or C) or autoimmune hepatitis, other untreated acute liver diseases, and advanced cirrhosis or hepatic failure requiring transplantation or emergency care. Patients with alcoholic hepatitis, hemochromatosis, Wilson’s disease, severe renal impairment, or those receiving anticoagulant therapy or drugs interfering with vitamin K metabolism were excluded. To minimize confounding effects on coagulation parameters, patients receiving anticoagulant or antiplatelet therapy were excluded. Subjects with advanced cardiovascular disease, recent myocardial infarction, or severe systemic comorbidities (e.g., metastatic cancer, autoimmune, or chronic pulmonary diseases) that could confound outcome assessment were also excluded. These criteria were applied to reduce potential confounding factors that could influence coagulation and metabolic parameters. All included patients received standard inpatient care according to institutional protocols.

### 2.4. Vitamin K Administration

All included patients received vitamin K supplementation during hospitalization as part of routine clinical management of coagulation abnormalities. A total of 65 patients (77.4%) received vitamin K intravenously at a daily dose of 10 mg, while 19 patients (22.6%) received oral vitamin K at a daily dose of 10 mg. The choice of administration route was based on clinical judgment and routine hospital practice rather than a standardized dosing protocol.

### 2.5. Laboratory and Clinical Parameters

Liver function and coagulation parameters were monitored during hospitalization. The analyzed laboratory parameters included aminotransferases, total bilirubin, cholinesterase, prothrombin time (PT), international normalized ratio (INR), and activated partial thromboplastin time (aPTT), along with selected metabolic indicators such as fasting glucose and lipid profile.

### 2.6. Statistical Analysis

Continuous data were presented as mean ± standard deviation (SD) or as median values with interquartile ranges (IQR: 25th and 75th percentiles), while categorical data were expressed as frequencies and percentages. Comparisons of laboratory parameters before and after vitamin K administration were performed using non-parametric tests (Friedman or Wilcoxon tests) [22,23]. A *p*-value < 0.05 was considered statistically significant.

### 2.7. Ethical Approval

The study was approved by the Scientific Research Ethics Committee of the “Lucian Blaga” University of Sibiu (approval no. 1/17 January 2025). All patient data were anonymized prior to analysis, and written informed consent for the use of medical data for research purposes was obtained from each participant at the time of hospitalization.

## 3. Results

### 3.1. Baseline Characteristics of the Study Population

A total of 84 patients diagnosed with metabolic dysfunction-associated steatohepatitis (MASH) who received vitamin K supplementation during hospitalization between January 2020 and December 2024 were included in the analysis. The mean age of the study population was 54.69 ± 15.28 years, and male patients represented 57.14% of the cohort. The distribution of residence was relatively balanced, with 46.43% of patients from urban areas and 53.57% from rural settings (Table 1).

The mean body mass index (BMI) was 28.89 ± 3.61 kg/m^2^, and the mean waist circumference was 107.58 ± 21.05 cm, consistent with abdominal obesity frequently observed in patients with metabolic liver disease. Fibrosis staging assessed by transient elastography showed that the largest proportion of patients were classified as stage F2 (40.47%), followed by F1 (25.00%), while advanced fibrosis stages (F3–F4) accounted for approximately one third of the cohort (Table 1).

A total of 65 patients (77.4%) received vitamin K intravenously at a daily dose of 10 mg, while 19 patients (22.6%) received oral vitamin K at the same daily dose. The mean duration of hospitalization was 8.92 ± 5.70 days. Most patients were discharged after clinical management (85.71%), while in-hospital mortality occurred in 14.29% of cases Table 1).

The baseline demographic and clinical characteristics of the study population are summarized in Table 1.

### 3.2. Changes in Coagulation Parameters

**Figure 1:**
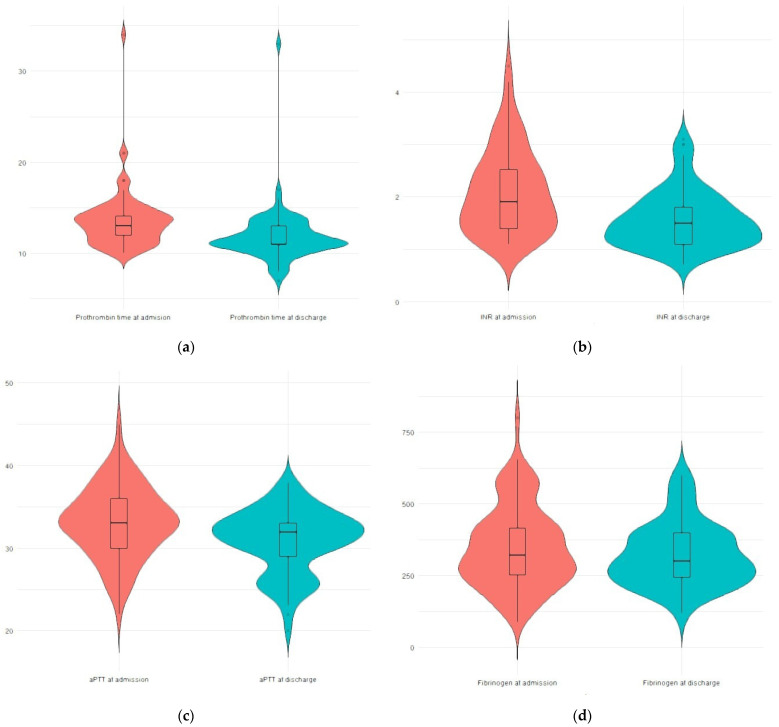
(**a**) Prothrombin time (PT) at admission and discharge; (**b**) international normalized ratio (INR) at admission and discharge; (**c**) activated partial thromboplastin time (aPTT) at admission and discharge; (**d**) Fibrinogen levels at admission and discharge.

Coagulation parameters demonstrated statistically significant changes during hospitalization. Prothrombin time (PT) decreased from a median of 13.00 s (IQR 12.00–14.00) at admission to 11.00 s (IQR 11.00–13.00) at discharge (*p* < 0.001). Activated partial thromboplastin time (aPTT) decreased from 33.00 s (IQR 30.00–36.00) to 32.00 s (IQR 29.00–33.00) (*p* < 0.001), while fibrinogen levels declined from 321 mg/dL (IQR 252–421) to 300 mg/dL (IQR 244–400) (*p* < 0.001) (Figure 1).

Although the median INR remained numerically similar between admission and discharge, a narrowing of the interquartile range was observed, indicating reduced variability and stabilization of coagulation values during hospitalization.

### 3.3. Changes in Hepatic and Metabolic Parameters

Liver enzyme levels showed modest numerical variations during hospitalization. Aspartate aminotransferase (AST) decreased from a median of 45.00 U/L (IQR 29.00–79.00) at admission to 41.00 U/L (IQR 21.00–76.50) at discharge (*p* < 0.001). Similarly, alanine aminotransferase (ALT) declined from 37.00 U/L (IQR 21.50–58.50) to 32.00 U/L (IQR 13.50–54.50) (*p* < 0.001).

Metabolic parameters also showed small numerical variations. Total cholesterol decreased slightly from 278.50 mg/dL (IQR 256.00–316.00) at admission to 275.50 mg/dL (IQR 245.50–299.50) at discharge (*p* < 0.001). Fasting glucose declined marginally from 91.00 mg/dL (IQR 79.50–168.00) to 90.00 mg/dL (IQR 77.00–184.00) (*p* < 0.001). Cholinesterase activity increased from 3599.50 U/L (IQR 2423.50–5484.00) to 3827.00 U/L (IQR 2499.50–7270.00) (*p* < 0.001).

### 3.4. Summary of Laboratory Changes

To facilitate direct comparison of laboratory parameters measured at admission and discharge, the main biochemical variables are summarized in Table 2.

### 3.5. Fibrosis Stage Subgroup Analysis

When comparing biochemical parameters across fibrosis stages (F0–F4), values showed numerical differences within relatively narrow ranges. Mean PT values at admission ranged from 12.00 s in F0 to 13.73 s in F4, and from 12.00 s to 11.40 s at discharge.

Mean INR ranged from 2.00 in F0 to 2.47 in F4 at admission and from 2.00 to 1.73 at discharge. Cholinesterase levels increased across fibrosis categories, with values ranging from 4000.00 U/L in F0 to 5026.94 U/L in F2 at admission and from 4200.00 U/L to 6094.15 U/L at discharge.

Fasting glucose values ranged from 89.00 mg/dL in F0 to 145.00 mg/dL in F4 at admission, and from 90.00 mg/dL to 117.47 mg/dL at discharge. Total cholesterol varied from 245.00 mg/dL in F0 to 304.14 mg/dL in F1 at admission, and between 201.00 mg/dL and 286.71 mg/dL at discharge.

Across all fibrosis categories, these variations remained numerical, and no statistically significant differences were identified between fibrosis groups (*p* > 0.05). To further explore fibrosis-related differences, patients were additionally grouped into non-significant fibrosis (F0–F2) and significant fibrosis (F3–F4), in accordance with current guidelines. No statistically significant differences were observed between the two groups in terms of coagulation or metabolic parameters.

## 4. Discussion

The present study describes short-term biochemical changes observed during hospitalization in patients with MASH receiving vitamin K supplementation. Our analysis revealed modest changes across several coagulation and metabolic parameters during hospitalization. Nevertheless, these findings provide valuable insights into the interplay between vitamin K status, hepatic function, and metabolic homeostasis in MASH, and they extend the existing literature by examining this relationship in a real-world hospitalized population. Below, we interpret these results in the context of published evidence and discuss potential mechanisms, clinical implications, and future research needs.

Importantly, the present study should be interpreted within the context of its retrospective and observational design. All patients were evaluated during hospitalization, a setting that inherently includes standardized medical care, temporary alcohol abstinence, dietary regulation, optimized glycemic management, rest, and treatment of intercurrent conditions. These factors are well known to influence liver enzymes, glucose levels, and lipid metabolism independently of any specific pharmacological intervention and likely had a significant impact on the observed biochemical changes; they should be considered major confounders when interpreting the results.

Consequently, the observed reductions in transaminases, fasting glucose, and total cholesterol cannot be attributed solely to vitamin K supplementation. Rather, these changes likely reflect the combined effect of standard inpatient care and metabolic stabilization during hospitalization, with vitamin K potentially acting as a supportive rather than causative factor. Changes in transaminases should be interpreted with caution, as they are nonspecific and may be influenced by factors other than MASH, including acute illness, metabolic fluctuations, or hospitalization-related interventions.

Short-term variations in metabolic parameters observed during hospitalization may also reflect the influence of hospitalization-related factors rather than a direct effect of vitamin K supplementation. In particular, alcohol cessation, hospital-controlled dietary intake, and structured medical management may rapidly influence serum glucose and lipid levels in patients with metabolic liver disease. Hospitalization-related factors, including dietary regulation, alcohol abstinence, route of drug administration, and standardized medical care, likely contributed to the observed biochemical variations and represent important confounders. The indication for hospitalization in this cohort was heterogeneous and may have included acute conditions that independently influenced coagulation parameters and liver enzyme levels. These factors likely contributed to the observed biochemical variations and represent important confounders that limit the interpretation of vitamin K-related effects.

An important aspect of our findings is the presence of abnormal coagulation parameters in patients with predominantly early-stage fibrosis (F0–F2). These abnormalities should not be interpreted as evidence of advanced liver disease, as they may reflect transient or multifactorial disturbances, including metabolic dysfunction, systemic inflammation, or acute illness during hospitalization.

Our study documented a consistent, although numerically modest, improvement in PT, INR, and aPTT between admission and discharge after vitamin K administration in patients with MASH, with no major differences across fibrosis stages. Taken together with the parallel rise in cholinesterase and the absence of clinically significant bleeding events, these findings support the hypothesis that a proportion of patients with metabolic liver disease may exhibit a functional vitamin K deficit superimposed on relatively preserved synthetic capacity. This profile differs from advanced cirrhosis, where repeated studies have shown that vitamin K administration rarely leads to meaningful INR correction and provides limited clinical benefit [24,25]. In contrast, our cohort—predominantly composed of patients with compensated disease—may represent a population in which vitamin K supplementation more readily translates into measurable biochemical effects.

An additional consideration in the interpretation of our findings relates to the limitations of conventional coagulation parameters in liver disease. It is well established that markers such as INR do not fully capture the complex balance between procoagulant and anticoagulant pathways and may not accurately reflect bleeding risk or overall hemostatic status. This limitation is particularly relevant in patients with early-stage liver disease, in whom alterations in INR may occur in the absence of advanced hepatocellular dysfunction. In this context, the modest changes observed in coagulation parameters in our cohort should be interpreted with caution, as they may not directly correspond to clinically meaningful changes in coagulation status [26]. These results should be interpreted in the broader context of heterogeneity in the literature regarding vitamin K responsiveness in chronic liver disease. Aldrich and Regal emphasized that empirical vitamin K administration in cirrhosis typically lacks efficacy and should not be used as routine correction prior to invasive procedures [24]. However, Xiong et al. observed that, in carefully selected patients with chronic liver failure, vitamin K1 supplementation was associated with both improved coagulation profiles and enhanced survival [27]. Our findings align with the latter hypothesis by suggesting that patients in earlier fibrotic stages—such as those with MASH—may retain sufficient hepatocellular function to benefit from vitamin K repletion. Nevertheless, the absence of statistically significant differences between fibrosis categories underscores the need for controlled prospective studies to better delineate the clinical impact of these biochemical changes.

Beyond coagulation, we noted concurrent reductions in transaminases, fasting glucose, and total cholesterol, along with increases in cholinesterase and stabilization of albumin. Although causality cannot be attributed solely to vitamin K in the context of a retrospective study, these trends are biologically plausible. Experimental research in high-fat-diet mouse models demonstrates that vitamin K2 reduces hepatic steatosis, visceral adiposity, and cholesterol accumulation [28], while human epidemiologic studies link higher vitamin K intake with a lower prevalence of metabolic-associated fatty liver disease (MASLD) and improved metabolic parameters [29]. Furthermore, randomized controlled trials in patients with type 2 diabetes have shown that vitamin K2 or K4 supplementation can improve markers of insulin sensitivity and lipid metabolism, despite modest effects on fasting glucose [30,31]. Within this framework, the metabolic improvements observed in our cohort likely reflect the combined influence of hospitalization-related factors and routine clinical care, rather than a specific therapeutic effect of vitamin K.

It is important to distinguish statistical significance from clinical relevance when interpreting the metabolic findings of this study. Although changes in fasting glucose and total cholesterol reached statistical significance, the absolute magnitude of these differences was minimal (median reductions of 1 mg/dL for glucose and 3 mg/dL for total cholesterol). Such changes are unlikely to translate into clinically meaningful metabolic benefits and should not be interpreted as a therapeutic effect.

These statistically significant results likely reflect the large sample consistency and paired analysis rather than a true metabolic impact of vitamin K supplementation. Therefore, the metabolic findings should be considered descriptive and exploratory, rather than indicative of clinical efficacy.

In addition to the coagulation and metabolic outcomes previously discussed, our stratified analysis across fibrosis stages (F0–F4) did not reveal statistically significant differences in the biochemical response to vitamin K administration. Although baseline values showed some expected gradients—such as slightly higher PT and INR and elevated fasting glucose in more advanced fibrosis—the overall trajectory of improvement from admission to discharge remained relatively uniform. This pattern may reflect a ceiling effect in hepatocellular synthetic impairment once fibrosis progresses beyond a certain threshold, limiting the potential for vitamin K-dependent pathways to fully normalize. Similar observations have been reported in dietary and micronutrient studies focusing on MASLD, where lower vitamin K intake correlates with disease presence, yet the association with fibrosis severity appears to weaken in advanced stages [29]. These findings support the interpretation that vitamin K supplementation may exert its greatest relative benefit earlier in the disease course, when hepatocyte function is still sufficiently preserved.

From a clinical management perspective, our results raise the possibility that assessing and correcting vitamin K status could be a valuable adjunctive strategy in patients with MASH, particularly in those exhibiting mild coagulopathy or metabolic instability. The numerical improvements we observed in fasting glucose, total cholesterol, and cholinesterase—although not statistically significant—suggest biological plausibility and align with epidemiologic evidence linking higher vitamin K intake to improved outcomes in MASLD. Notably, analyses from large population datasets have demonstrated that greater dietary vitamin K consumption is associated with reduced all-cause mortality in patients with NAFLD [29], while national cohort studies indicate that both macro- and micronutrient composition, including vitamin K, may influence MASLD progression and metabolic phenotype [32]. Within this context, vitamin K supplementation should not be viewed as a standalone therapy, but rather as a complementary intervention integrated into broader metabolic and hepatologic care.

An additional aspect relevant to the interpretation of our findings concerns the interplay between vitamin K status, dietary intake, and metabolic dysfunction in MASLD/MASH. Several population-based studies have demonstrated that individuals with MASLD tend to have significantly lower habitual vitamin K intake compared with metabolically healthy controls, suggesting a potential nutritional contribution to the pathogenesis of fatty liver disease [16]. Furthermore, higher dietary vitamin K intake has been associated with reduced all-cause mortality in NAFLD patients and with more favorable metabolic profiles, supporting the notion that vitamin K availability may modulate both hepatic and systemic metabolic pathways [29,32]. Within this context, the biochemical variations observed in our cohort—particularly in fasting glucose, total cholesterol, and cholinesterase—may reflect changes occurring during hospitalization rather than a specific metabolic effect of vitamin K supplementation.

Another important consideration is the variability of vitamin K absorption and bioavailability across different forms and stages of liver disease. In cholestatic or decompensated conditions, impaired intestinal absorption of fat-soluble vitamins frequently leads to clinically relevant vitamin K deficiency, which often necessitates parenteral replacement rather than oral administration [17]. In contrast, the patients included in our study predominantly had compensated MASH without overt cholestasis, a scenario in which the standard administration of vitamin K is expected to be more efficient and biologically meaningful. This may explain why we observed measurable improvements in coagulation and metabolic markers, whereas studies in critically ill patients or advanced liver disease have reported high rates of persistent vitamin K deficiency, elevated protein induced by vitamin K absence or antagonism II PIVKA-II levels, and limited biochemical response despite supplementation [33]. These considerations highlight the need for future prospective studies to stratify patients not only by fibrosis stage but also by cholestatic features, nutritional status, and baseline vitamin K-dependent biomarkers in order to better characterize which subgroups derive the most benefit.

This study has several limitations that should be acknowledged when interpreting the results. Its retrospective and observational design precludes establishing causality between vitamin K administration and the observed biochemical changes, particularly as all patients received multimodal treatment during hospitalization. Factors such as glycemic control optimization, dietary adjustments, management of intercurrent infections, or temporary abstinence from alcohol may have independently influenced the metabolic and coagulation parameters assessed. The lack of standardized dosing protocols and variability in pharmacologic regimens further introduces heterogeneity that may confound the response to vitamin K. In addition, vitamin K status was not objectively measured through plasma vitamin K or PIVKA-II levels, making any presumed deficiency inferential rather than confirmed. The relatively small sample size, single-center nature, and heterogeneous distribution of comorbidities and fibrosis stages limit generalizability and reduce the ability to perform meaningful subgroup analyses, particularly for advanced fibrosis categories. A major limitation of this study is the absence of a control group of hospitalized MASH patients who did not receive vitamin K supplementation. As a result, it is not possible to isolate the specific contribution of vitamin K from the general effects of hospitalization, including dietary interventions, enforced abstinence, rest, and comprehensive medical management. Additionally, the retrospective nature of the study precludes causal inference. Therefore, the findings should be considered hypothesis-generating and interpreted as descriptive of the short-term biochemical course of hospitalized MASH patients receiving vitamin K as part of routine care. Another important limitation is that vitamin K status was not objectively assessed, as neither PIVKA-II nor serum vitamin K levels were measured. Therefore, it cannot be determined whether patients had a true functional vitamin K deficiency or whether vitamin K supplementation was administered empirically. In the context of MASH, where intestinal absorption of fat-soluble vitamins is often preserved compared with cholestatic liver disease, the observed changes in coagulation parameters may reflect non-specific effects rather than correction of a documented deficiency. Another limitation relates to the heterogeneity of diagnostic methods used to assess liver fibrosis. In this retrospective cohort, fibrosis staging was based on a combination of non-invasive modalities, including imaging techniques and serum-based tests such as FibroTest and transient elastography. These approaches differ in diagnostic performance and cut-off values, and the absence of a unified diagnostic standard—such as liver biopsy or a standardized elastography protocol—may reduce the precision of fibrosis stratification in the present analysis. An additional limitation of the fibrosis subgroup analysis relates to the uneven distribution of patients across fibrosis stages. In particular, the small number of cases in the F0 and F3 categories compared with the larger F2 group may have reduced the statistical power to detect potential differences between fibrosis stages. Finally, the study focused exclusively on short-term biochemical outcomes and did not evaluate long-term clinical endpoints such as bleeding events, fibrosis progression, or survival. Therefore, the findings should be interpreted as descriptive of in-hospital biochemical dynamics rather than as evidence of a direct pharmacological effect of vitamin K. These minimal numerical variations fall within the range of expected biological fluctuations and should not be interpreted as therapeutic effects. Unlike our previous analysis performed in patients with advanced cirrhosis, the present study focuses on a predominantly non-cirrhotic MASH population, allowing the exploration of vitamin K-related biochemical responses in earlier disease stages, where hepatocellular function is relatively preserved.

The present study should be considered hypothesis-generating and intended to inform the design of future prospective controlled studies. Future studies should define more clearly which patients with MASH benefit from vitamin K supplementation and under what conditions it is most effective. Prospective trials with standardized dosing and direct measures of vitamin K status are needed to confirm true treatment responsiveness. In addition, evaluating the influence of fibrosis stage and cholestatic features, along with assessing long-term clinical outcomes, will be essential to determine the real therapeutic value of vitamin K in this population.

## 5. Conclusions

Vitamin K supplementation was associated with modest changes in coagulation parameters and small, clinically negligible variations in selected metabolic markers in patients with MASH, irrespective of fibrosis stage. These findings describe biochemical changes observed in hospitalized patients receiving vitamin K, although the magnitude of metabolic variations was small. Although the magnitude of these changes is limited, the results indicate that vitamin K could serve as a supportive component within individualized management strategies. These findings describe short-term biochemical changes observed during hospitalization and should not be interpreted as evidence of a causal or clinically meaningful therapeutic effect. Further prospective studies are needed to determine the clinical relevance of vitamin K administration in this population.

## Figures and Tables

**Table 1:** Baseline characteristics of the study population.

Age (years, mean ± SD)	54.69 ± 15.28
Sex (male/female *n*(%))	48 (57.14%)/36 (42.86%)
Residence (urban/rural *n*(%))	39 (46.43%)/45 (53.57%)
BMI (kg/m^2^, mean ± SD)	28.89 ± 3.61
Fibrosis stages (*n* (%))	
F0	1 (1.19%)
F1	21 (25.00%)
F2	34 (40.47%)
F3	13 (15.47%)
F4	15 (17.84%)
Hospitalization (days, mean ± SD)	8.92 ± 5.70
Death/Discharged (*n*, %)	12 (14.29%)/72 (85.71%)

**Table 2:** Laboratory parameters at admission and discharge.

Laboratory Characteristics	Admission	Discharge	*p*-Value
PT (s)	13.00 (12.00–14.00)	11.00 (11.00–13.00)	0.000
INR	2.00 (1.00–2.25)	2.00 (1.00–2.00)	0.000
aPTT (s)	33.00 (30.00–36.00)	32.00 (29.00–33.00)	0.000
AST (U/L)	45.00 (29.00–79.00)	41.00 (21.00–76.50)	0.000
ALT (U/L)	37.00 (21.50–58.50)	32.00 (13.50–54.50)	0.000
Total Cholesterol (mg/dL)	278.50 (256.00–316.00)	275.50 (245.50–299.50)	0.000
Glucose (mg/dL)	91.00 (79.50–168.00)	90.00 (77.00–184.00)	0.000
Fibrinogen (mg/dL)	321.00 (252.00–421.00)	300.00 (244.00–400.00)	0.000

PT—prothrombin time, INR—international normalized ratio, aPTT—activated partial thromboplastin time, AST—aspartate aminotransferase, ALT—alanine aminotransferase.

## Data Availability

The original contributions presented in this study are included in the article. Further inquiries can be directed to the corresponding authors.

## Abbreviations

The following abbreviations are used in this manuscript:

APTT/aPTT	Activated partial thromboplastin time
AST	Aspartate aminotransferase
ALT	Alanine aminotransferase
BMI	Body mass index
CT	Computed tomography
IQR	Interquartile range
INR	International normalized ratio
MASLD	Metabolic dysfunction-associated steatotic liver disease
MASH	Metabolic dysfunction-associated steatohepatitis
MRI	Magnetic resonance imaging
NAFLD	Non-alcoholic fatty liver disease
NASH	Non-alcoholic steatohepatitis
PT	Prothrombin time
TC	Total cholesterol
TG	Triglycerides
T2DM	Type 2 diabetes mellitus
US	Ultrasound
